# Prevalence of urinary tract infections and risk factors among diabetic patients in Ethiopia, a systematic review and meta-analysis

**DOI:** 10.1371/journal.pone.0278028

**Published:** 2023-01-17

**Authors:** Kirubel Dagnaw Tegegne, Gebeyaw Biset Wagaw, Natnael Atnafu Gebeyehu, Lehulu Tilahun Yirdaw, Nathan Estifanos Shewangashaw, Mesfin Wudu Kassaw

**Affiliations:** 1 Department of Comprehensive Nursing, College of Medicine and Health Science, Wollo University, Dessie, Ethiopia; 2 Department of Pediatrics and Child Health Nursing, College of Medicine and Health Science, Wollo University, Dessie, Ethiopia; 3 Department of Midwifery, College of Medicine and Health Science, Wolita Sodo University, Wolita Sodo, Ethiopia; 4 Department of Emergency Nursing, College of Medicine and Health Science, Wollo University, Dessie, Ethiopia; 5 School of Nursing, College of Health Science, Woldia University, Woldia, Ethiopia; Gulf Medical University, UNITED ARAB EMIRATES

## Abstract

**Introduction:**

Urinary tract infection (UTI) is a common clinical problem that comprises 1–6% of medical referrals and includes urinary tract, bladder, and kidney infections. UTI is the most commonly occurring infectious disease in diabetic patients. Therefore, this systematic review and meta-analysis aimed to estimate the prevalence of urinary tract infection and its associated factors in Ethiopia.

**Methods:**

The online libraries of PubMed, Google Scholar, Scopus, and Science Direct, were searched. Data were extracted using Microsoft Excel and analyzed using STATA statistical software (v. 16). Forest plots, Begg’s rank test, and Egger’s regression test were all used to check for publication bias. To look for heterogeneity, I^2^ was computed, and an overall estimated analysis was carried out. Subgroup analysis was done by region, and publication year. Meta-regression analysis using study-level covariates as predictors of study-level estimates to explore the determinants of potential heterogeneity in our pooled estimates. The pooled odds ratio for related covariates was also calculated.

**Results:**

Out of 1128 studies assessed, 14 met our criteria and were included in the study. A total of 3773 people were included in the study. The prevalence of urinary tract infection was estimated to be 15.97% (95% CI: 12.72–19.23). According to subgroup analysis, the highest prevalence was observed in the SNNP region (19.21%) and studies conducted in and after 2018 (17.98%). Being female (AOR = 3.77; 95% CI: 1.88, 5.65), being illiterate (AOR = 5.29; 95% CI: 1.98, 8.61), prior urinary tract infection history (AOR = 3.04; 95% CI: 2.16–3.92) were the predictor of urinary tract infection.

**Conclusion:**

The prevalence of urinary tract infections was high in Ethiopia. Female gender, illiteracy, and prior UTI history were associated with urinary tract infections. Since UTIs in diabetic patients has serious medical and public health consequence, screening of UTIs in diabetic patients and early initiation of treatment should become a public health priority.

## Introduction

Diabetes is the most common type of endocrine disorder in the last century. In developing countries, a variety of factors, including lifestyle changes, have contributed to the high occurrence of diabetes [1]. The prevalence of diabetes has increased in recent years [2].

To date, the International diabetic federation estimated that around 451 million people live with diabetes worldwide in 2017 and this number is projected to reach 693 million by 2045 if no prevention methods are implemented [3]. Around 79% of diabetes in the world live in low and middle-income countries [4]. Diabetes increases the occurrence of various diseases, such as cardiovascular diseases, eye and blindness problems, lower leg amputation, kidney diseases and infectious diseases [5–9]. Poor control of blood glucose in diabetic patients increases the chances of getting infections; therefore these individuals need to be cautious in preventing these infections more than the general population [10, 11]. Urinary tract infection (UTI) is a common clinical problem that comprises 1–6% of medical referrals and includes urinary tract, bladder, and kidney infections [12]. UTI is the most commonly occurring infectious disease in diabetic patients [13]. UTIs and its related complications result in about 150 million deaths a year around the globe [14].

A urinary tract infection (UTI) is an infection that grows within the urinary tract. This type of infection can involve the urethra (a condition known as urethritis), and bladder (a condition called cystitis) or may extend into the kidneys (a condition known as pyelonephritis) [13, 15, 16]. Women are more prone to develop UTIs than men, and infection confined to the bladder can be very painful. However, serious consequences of UTI occurs when the infection spreads to the kidneys [13, 15].

Factors such as, impaired immune system disorder, weakening of white blood cells, poor blood supply, bladder dysfunction due to nephropathy and glycosuria can cause urinary tract infections in diabetic patients [17–22]. Diabetic patients may experience dysuria as a complication of UTI due to organ damage and potentially life threatening conditions appear due to pyelonephritis and related kidney damage. Urinary tract infections make blood glucose control difficult in diabetics, which increases the need for continuous blood glucose monitoring, reducing patient’s quality of life, and impose significant healthcare burden in terms of cost [23].

Reports from various studies indicate heterogeneity in reported prevalence, indicating the inconsistency and uncertainty of the prevalence of UTI in diabetes patients. To the best of our knowledge, there is no published systematic reviews and meta-analysis study that estimates the prevalence of UTI and the risk factors. Therefore, since intervention studies on reducing the prevalence of UTI in diabetes patients require accurate and consistent information to prevent the complications of UTI, the research question is what is the overall prevalence of UTI in diabetes patients? The findings from this study could provide a better understanding of the condition and development of more detailed programs to reduce the effects of urinary tract infections and improve people’s health.

## Methods

This systematic review and meta-analysis study was conducted to determine the pooled prevalence of urinary tract infections and its determinants in Ethiopia using the standard PRISMA checklist guideline [24] (S1 Checkslist). This systematic review and meta-analysis is registered on PROSPERO under a registration number of CRD42022353520.

### Search strategy

International online databases (Pub Med, Science Direct, Scopus, and Google Scholar) were used to search and find a variety of articles on the prevalence of UTIs. The search string was established using "AND" and "OR" Boolean operators. Keywords were extracted from the Medical Subject Headings (MeSH) database. Keywords related to the studied population (P) were: prevalence, outbreak, diabetes and outcome-related keywords (O) were: UTI, urinary tract infection, infection, morbidity, outcomes. The following core search terms and phrases with Boolean operators were used to search related articles: (((((((((((prevalence) OR (magnitude)) AND (urinary tract infection)) OR (UTI)) OR (infection) AND (factors)) OR (associated factors)) OR (risk factors)) OR (determinants)) AND (diabetes)) OR (diabetes millets)) AND (Ethiopia). Therefore, all possible related articles published from January 1993 to May 2022 were identified, and their information was transferred to EndNote. In order to maximize the comprehensiveness of the search, the list of sources used in all related articles found in the above search was manually reviewed.

### Inclusion and exclusion criteria

This meta-analysis includes studies that reported the prevalence of UTI in diabetic patients as a study participants, only English language publications, both published and unpublished studies with full text available for search, and studies that took place in Ethiopia. Those studies that reported duplicated sources, qualitative studies from developed countries, and articles without full text available were excluded from this systematic review and meta-analysis.

### Quality assessment

Two authors (KDT and NAG) independently appraised the standard of the studies using the well-established Joanna Briggs Institute (JBI) standardized quality appraisal checklist for prevalence studies [25]. The score ranges from 0 to 9, with higher values corresponding to higher study quality (and less risk of bias). The disagreement raised during the quality assessment was resolved through a discussion led by the third author (GBW). Finally, the argument was solved and reached with an agreement. The critical analysis checklist has eight parameters with yes, no, unclear, and not applicable options. The parameters involve the following questions:

1. Where were the criteria for inclusion in the sample clearly defined?
2. Were the study subjects and, therefore, the setting described in detail?
3. Was the exposure measured result validly and reliably?
4. Were the main objective and standard criteria used to measure the event?
5. Were confounding factors identified?
6. Were strategies to affect confounding factors stated?
7. Were the results measured indeed and dependably? And, was the statistical analysis suitable? According to JBI standardized quality appraisal checklist, studies were considered low risk when they scored 50% and above on the quality assessment indicators, as reported in a S1 Table.

### Risk of bias assessment

Two authors (KDT and NAG) independently assessed included studies for risk of bias through the bias assessment tool developed by Hoy et al. [26], consisting of ten items that assess four domains of bias and internal and external validity. Any disagreement raised during the risk of bias assessment was resolved through a discussion led by the third author (GBW). Finally, the argument was solved and reached with an agreement. The first four items (items1–4) evaluate the presence of selection bias, non-response bias, and external validity. The other six items (items 5–10) assess the presence of measuring bias, analysis-related bias, and internal validity. Therefore, studies that received ’yes’ for eight or more of the ten questions were classified as ’low risk of bias.’ If studies that received ’yes’ for six to seven of the ten questions were classified as ’moderate risk’ whereas studies that received ’yes’ for five or fewer of the ten questions were classified as ’high risk’ as reported in a S2 Table.

### Data extraction

Microsoft Excel spreadsheet (2016) and STATA version 16 software were utilized for data extraction and analysis. Two authors (KDT and GBW) independently extracted all relevant data using a standardized Joanna Briggs Institute data extraction format. The disagreement raised during data extraction was resolved through a discussion led by the third author (LTY). Finally, the argument was solved and reached with an agreement. The data automation tool was not used due to this study’s absence of the paper form (manual data). The name of the first author, year of publication, study region, study setting, study design, the prevalence of urinary tract infection, and sample size was extracted.

### Data analysis

After extracting all relevant findings in a micro-soft excel spreadsheet, the data were exported to STATA software version 16 for analysis. The pooled prevalence of urinary tract infections was computed using a 95% confidence interval. Publication bias was checked by funnel plot and more objectively through Begg and Egger’s regression tests [27], and *p*-value less than 0.05 indicates it is statistically significant [28]. The presence of between-study heterogeneity was checked by using the Cochrane Q statistic. This heterogeneity between studies was quantified using I^2^, in which a value of 25, 50, and 75% represented low, medium, and high heterogeneity, respectively [29]. We used forest plot was used to visually assess the presence of heterogeneity, which presented at a high-level random-effect model was used for analysis to estimate the overall prevalence of UTI. Subgroups were performed according to region and publication year (<2018/≥2018). Sensitivity analysis was executed to examine the effect of a single study on the overall prevalence of the meta-analysis estimate. Furthermore, we performed meta-regression analysis using study-level covariates as predictors of study-level estimates to explore the determinants of potential heterogeneity in our pooled estimates. The findings of the study were presented in the form of text, tables, and figures.

## Results

**Fig 1:**
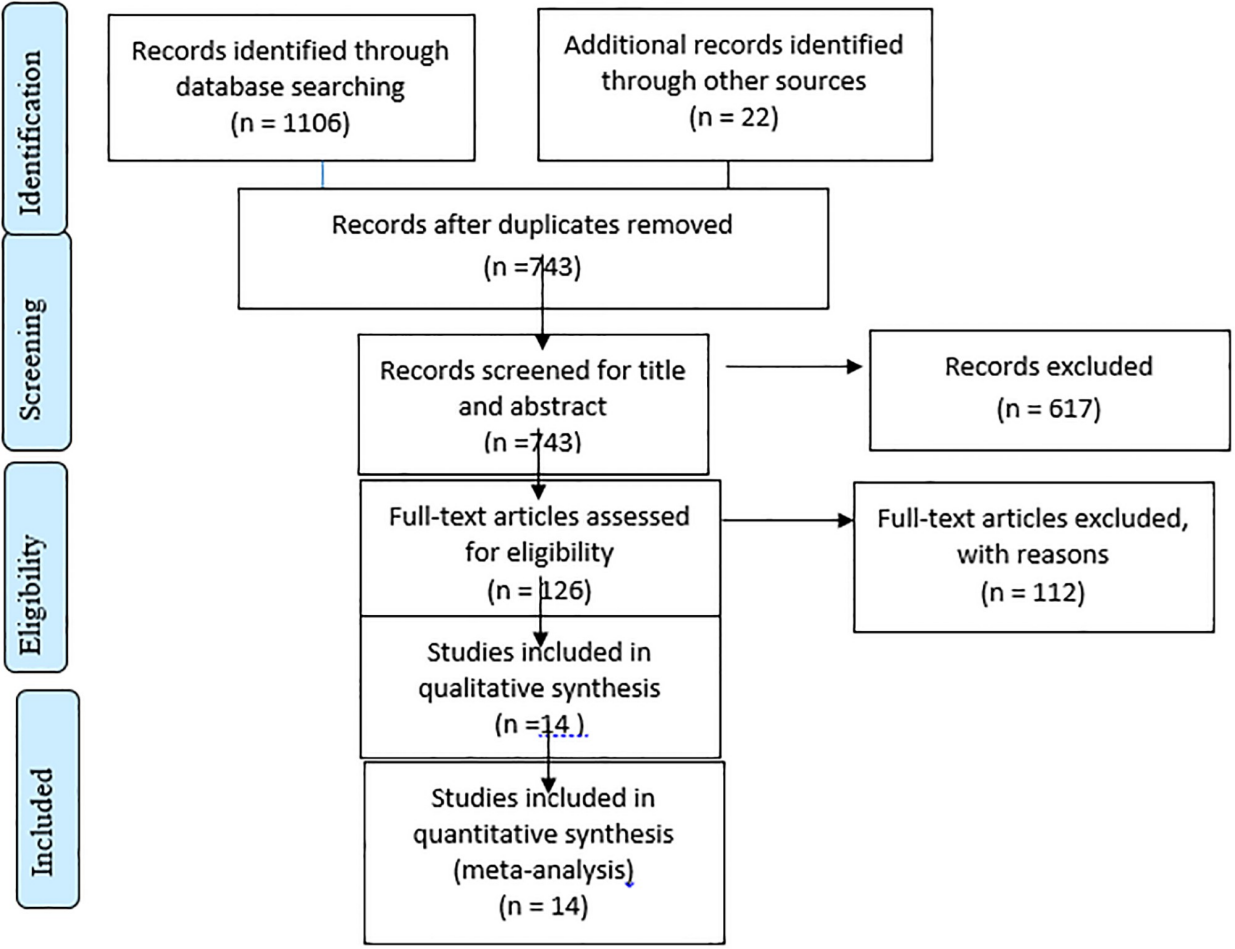
Flow chart illustrating the process of search and selection of studies included in the present systematic review and meta-analysis.

One thousand one hundred twenty-eight articles were retrieved using a search strategy about prevalence of urinary tract infection and associated factors in Ethiopia through online search engines such as; PubMed, Scopus, Google Scholar, Science direct, and online research repository home. After duplicates were removed, 743 articles remained. Then, 617 studies were excluded after reviewing for full title and abstracts from the remaining 743 studies. Therefore, 126 full-text studies were assessed for eligibility criteria, which further excluded 112 studies due to reasons. Finally, 14 articles were included as criteria for this systematic review and meta-analysis study (Fig 1).

### Characteristics of included studies

All included studies employed a cross-sectional study design. Besides, all the included studies was conducted in institutional bases. Four in Addis Ababa [30–33], Four in Amhara [34–37], Three in Southern Nations Nationalists and Peoples Region (SNNP) [38–40], two in Oromia [41, 42], and one study in Harari [43]. All studies in this review adopted a cross-sectional design. Sample sizes within the studies ranged from 179 to 422. The maximum (33.8%) and minimum (9.8%) prevalence rates of UTI appeared in the studies by Mohammed A. et al. [39] and Gebremedhin Y. et al. [32], respectively. All studies were assessed by using Joanna Briggs Institute (JBI) quality appraisal checklist and yielded low risk (Table 1).

**Table 1:** Summary characteristics of studies included in the meta-analysis.

Author	Year	Region	Setting	Study design	Sample size	Prevalence	Quality
Yeshitela. B et al. [33]	2012	Addis Abeba	Institutional	Cross-sectional	413	10.9	Low-risk
Feleke Y. et al. [30]	2007	Addis Abeba	Institutional	Cross-sectional	179	14	Low-risk
Walelgn B. et al. [35]	2021	Amhara	Institution	Cross-sectional	359	22.3	Low-risk
Abate D. et al. [43]	2017	Harari	Institutional	Cross-sectional	240	15.4	Low-risk
Nigussie D. et al. [40]	2017	SNNP	Institutional	Cross-sectional	240	13.8	Low-risk
Worku GY. et al. [32]	2021	Addis Abeba	Institutional	Cross-sectional	225	9.8	Low-risk
Yismaw G. et al. [37]	2012	Amhara	Institutional	Cross-sectional	422	17.8	Low-risk
Woldemariam HK. et al. [31]	2019	Addis Abeba	Institutional	Cross-sectional	248	22.6	Low-risk
Alemu M. et al. [34]	2020	Amhara	Institutional	Cross-sectional	336	11.6	Low-risk
Mama M. et al. [38]	2019	SNNP	Institutional	Cross-sectional	239	33.8	Low-risk
Worku S. et al. [36]	2017	Amhara	Institutional	Cross-sectional	192	10.9	Low-risk
Regea D. et al. [42]	2017	Oromia	Institutional	Cross-sectional	200	16.5	Low-risk
Gutema T. et al. [41]	2018	Oromia	Institutional	Cross-sectional	233	16.7	Low-risk
Mohammed A. et al. [39]	2020	SNNP	Institutional	Cross-sectional	247	10.5	Low-risk

### Meta-analysis

#### Prevalence of urinary tract infections in Ethiopia

**Fig 2:**
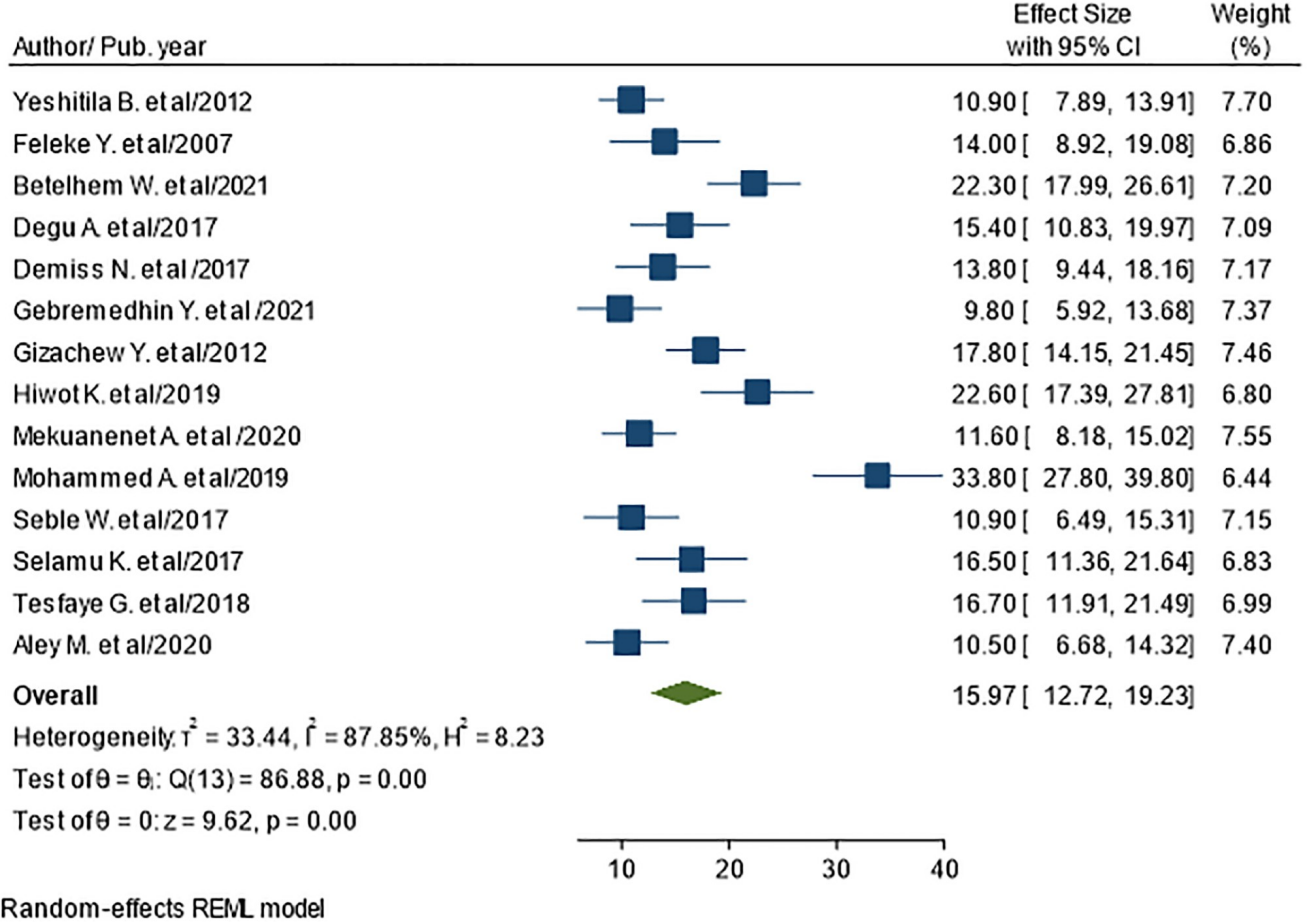
The pooled prevalence of UTI among diabetic populations in Ethiopia.

The pooled prevalence of urinary tract infections in Ethiopia are presented by the forest plots in (Fig 2). A random-effect model showed that the pooled prevalence of urinary tract infection was 15.97% (95% CI: 12.72–19.23; I^2^ = 87.85%).

### Sub-group analysis by region

**Fig 3:**
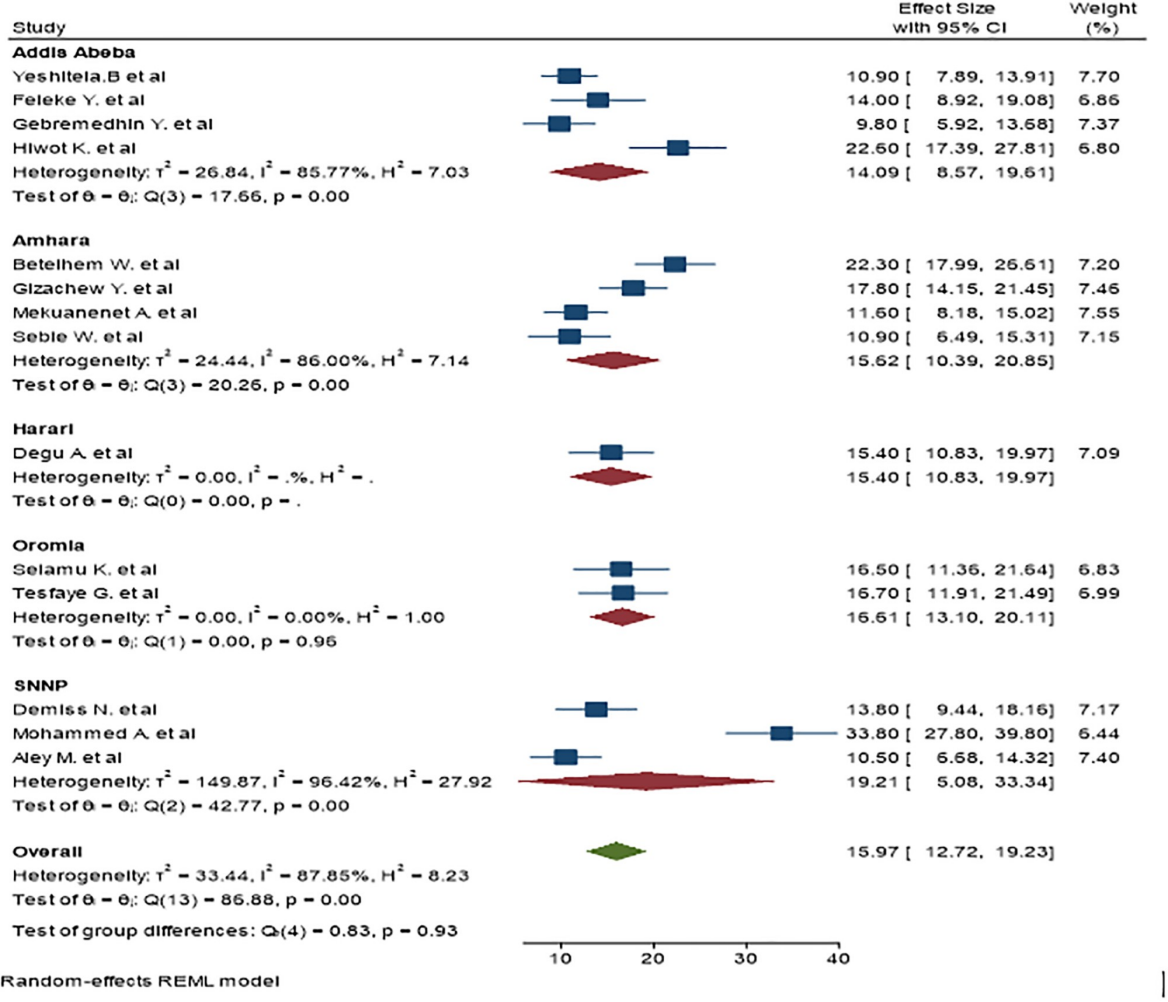
The pooled prevalence of UTI among diabetic patients based on region.

We observed high heterogeneity between studies (I^2^ = 87.85%). As a result, sub-group analysis is conducted based on study region. As a result, the highest pooled prevalence of urinary tract infection was in SNNP, 19.21% ((95% CI: 5.08–33.34) I^2^ = 96.42%, P<0.01 and the lowest was in Addis Ababa, 14.02% ((95% CI: 8.57, 19.61) I^2^ = 85.77%, P<0.01 (Fig 3).

### Sub-group analysis by year of publication

**Fig 4:**
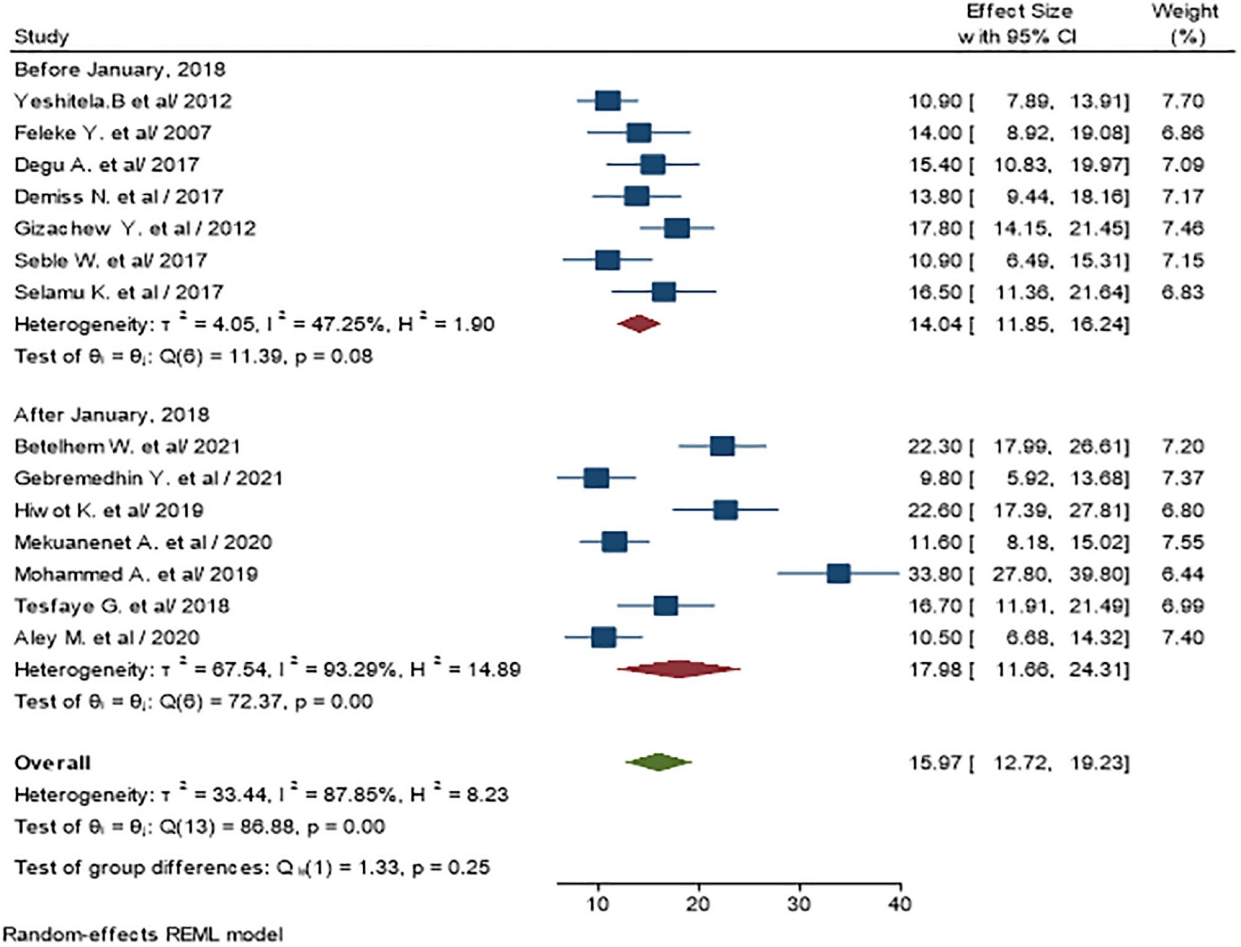
The pooled prevalence of urinary tract infections among diabetic patients based on publication year.

The pooled prevalence of urinary tract infection among diabetic patients was found to be 14.04% ((95% CI: 11.85, 16.24) I^2^ = 47.25%, P<0.01 on studies published before January 2018 and 17.98% ((95% CI: 11.66, 24.31) I^2^ = 93.29%, P<0.01 on studies published after January 2018 (Fig 4).

### Meta regression

To identify the sources of heterogeneity meta-regression was performed using year of publication and sample size as a covariate. Thus, it was indicated that there is no effect of year of publication and sample size on heterogeneity between studies as indicated by insignificant p-value (Table 2).

**Table 2:** Meta-regression analysis of factors affecting between-study heterogeneity.

Heterogeneity source	Coefficients	Std. Err.	P-value
Publication year	0.2570	0.4685	0.583
Sample size	0.0031	0.0233	0.892

### Publication bias

**Fig 5:**
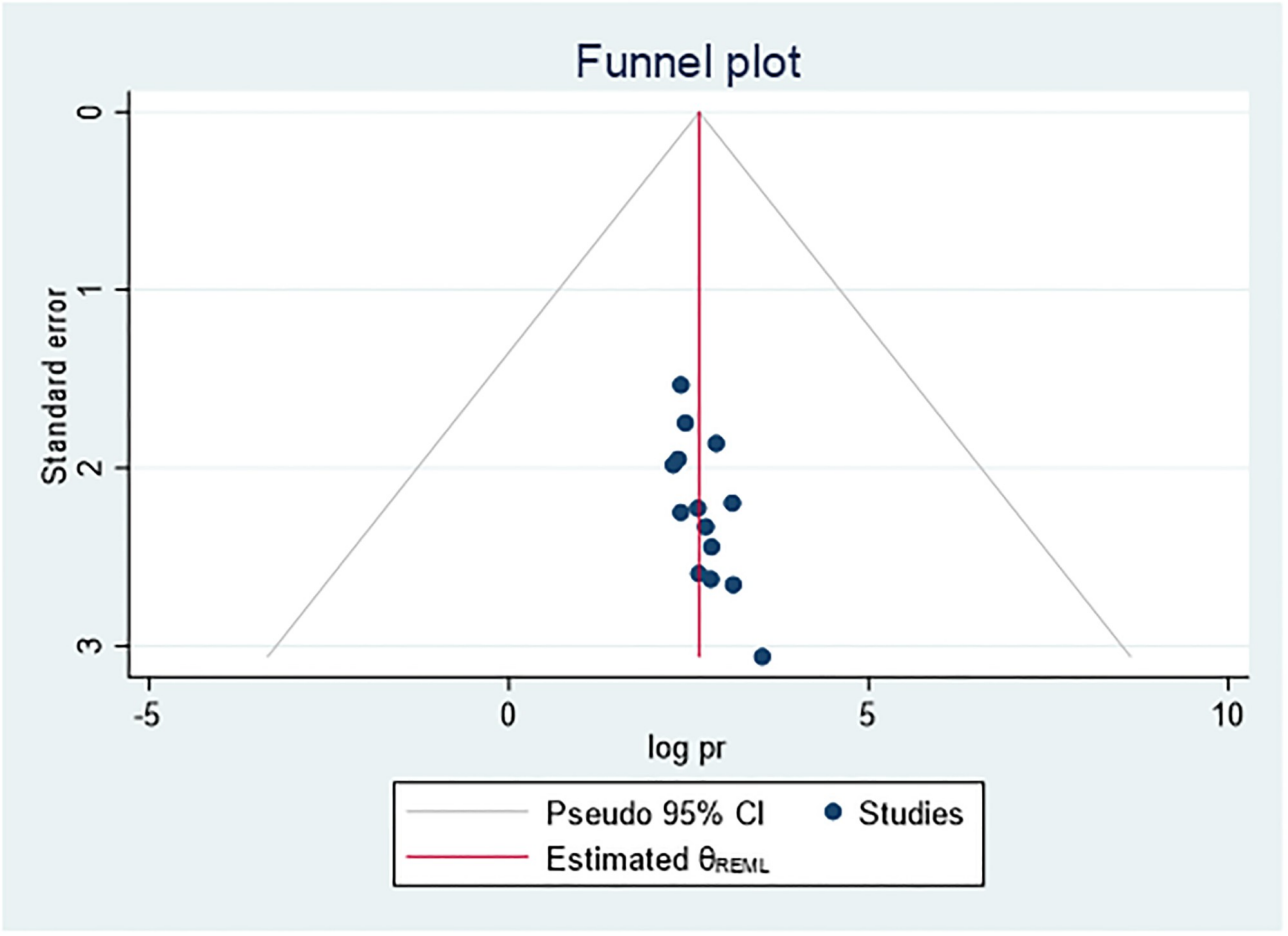
Funnel plot which shows symmetrical distribution.

**Fig 6:**
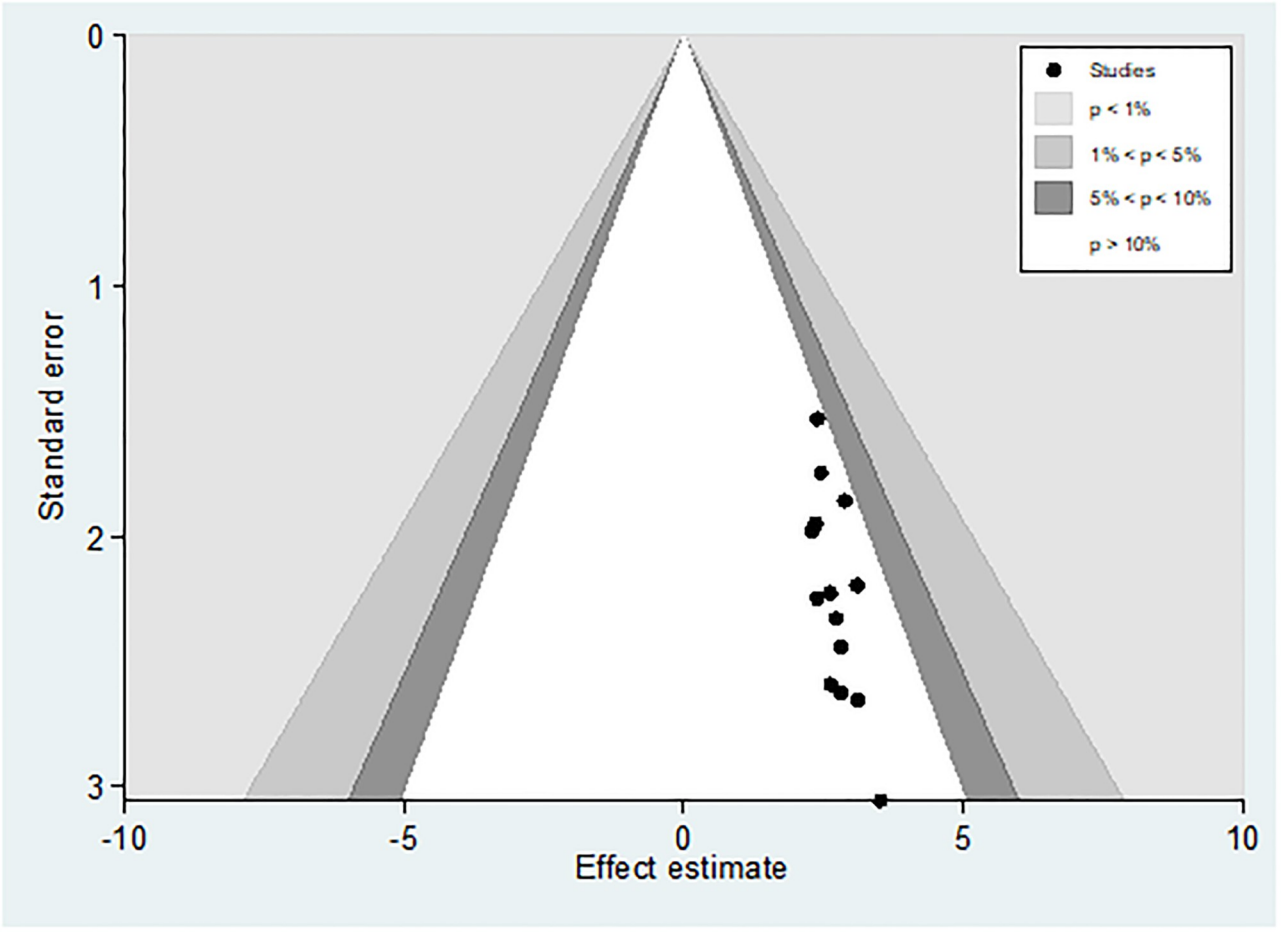
The trim-and-fill method was used to correct the result of one potentially missing study was required in the right side of the funnel plot to ensure symmetry.

**Fig 7:**
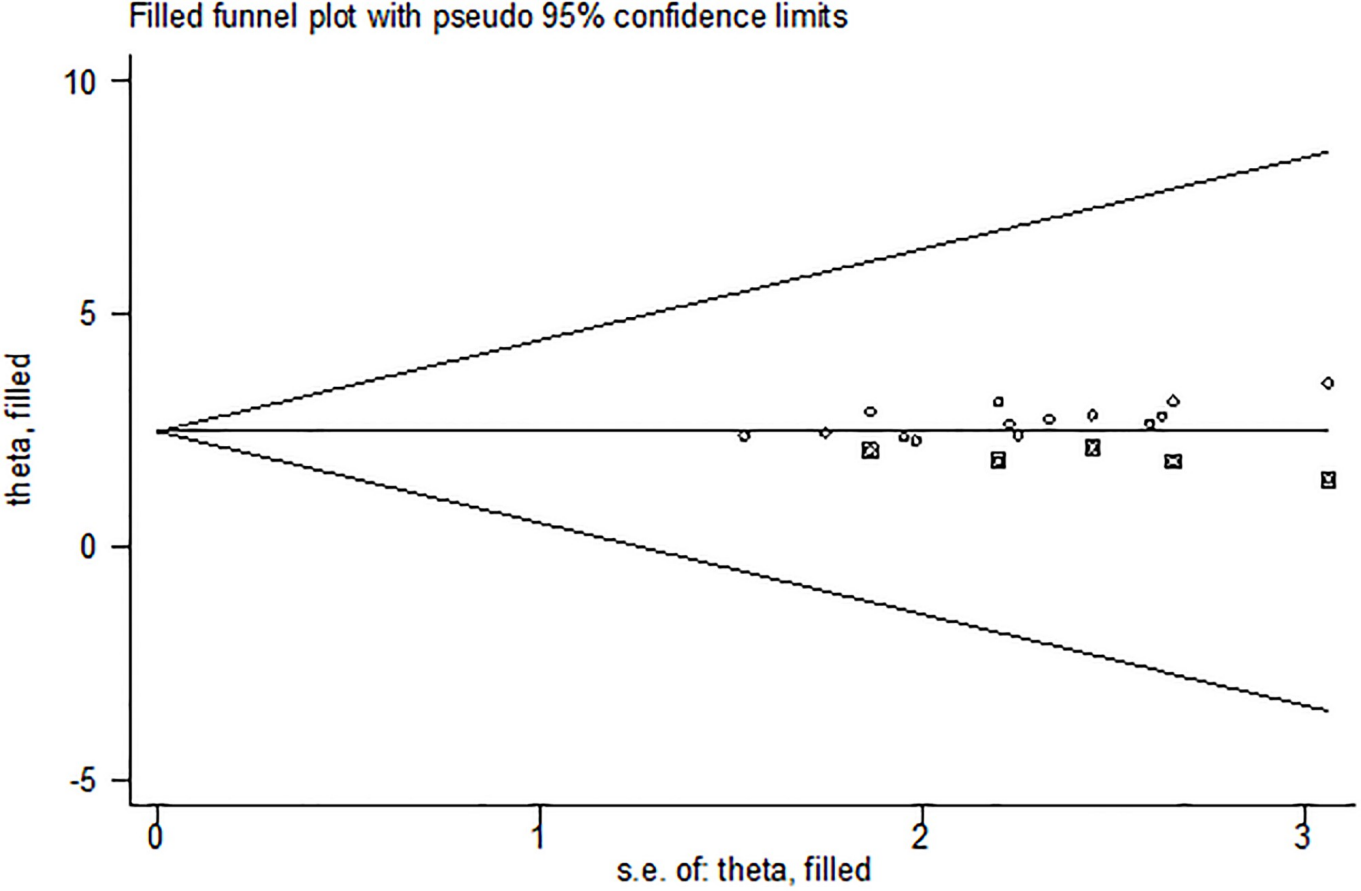
Counter enhanced funnel plot suggestions of missing studies on the bottom left-hand of the plot.

We attempted to minimize publication bias in this review in several ways. A comprehensive search has been made consulting with experts to identify grey or unpublished literature. Four major databases were searched. Reference lists of articles were screened to identify potentially eligible studies. At least two review authors independently scrutinized and selected articles for inclusion using pre-specified eligibility criteria, assessed risk of bias and extracted data. The funnel plot of the data shows symmetrical distribution of the studies from the line of effect (Fig 5). However, the result of Eggers (0.0006) and begs test (0.0118) show the presence of publication bias. Subsequently, trim-and-fill analysis was performed and indicated the presence of one unpublished study (Fig 6). A counter-enhanced funnel plot was also calculated, and the missing studies in the areas of higher statically significance suggested that the cause of asymmetry was due to factors other than publication bias, such as the study variables (Fig 7).

### Sensitivity analysis for the studies included in urinary tract infection

**Fig 8:**
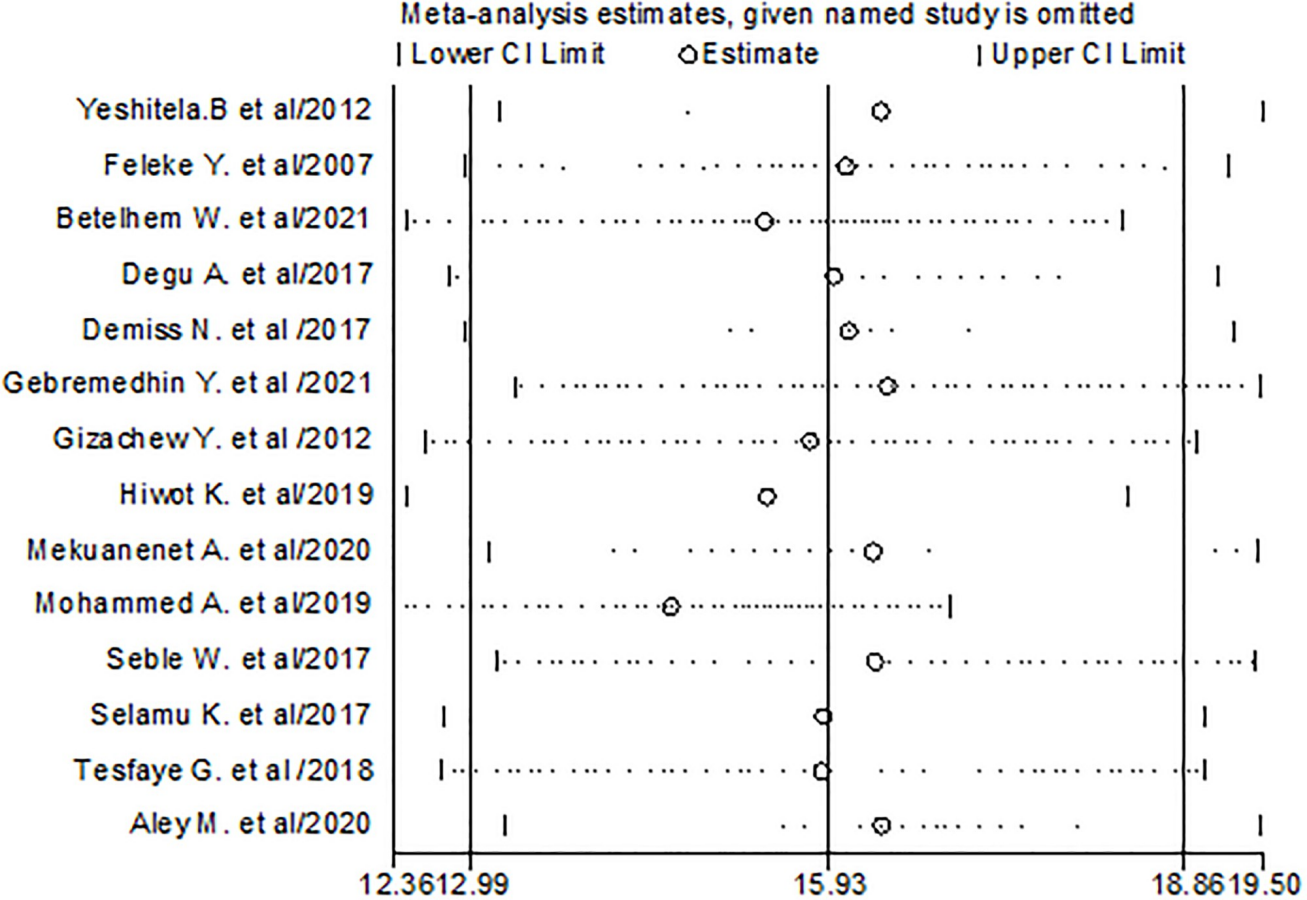
Results of sensitivity analysis of the 14 studies in the meta-analysis of UTI.

To check the individual effect of included studies on the pooled prevalence of urinary tract infection in Ethiopia, sensitivity analysis was performed using random effect model and the result revealed that there is no single study influenced on the pooled prevalence of UTI among diabetes. The pooled estimated prevalence of UTI is estimated from 14.65(12.36, 16.94) to 16.42(13.35, 19.48) after omission of a single study (Fig 8).

### Factors associated with urinary tract infections among diabetics

In this systematic review and meta-analysis we identified a variety of risk factors associated with UTI in diabetic patients. As a result, being female, having previous history of UTI, and being illiterate are the factors associated with UTI among diabetic patients.

### Association of sex with UTI

**Fig 9:**
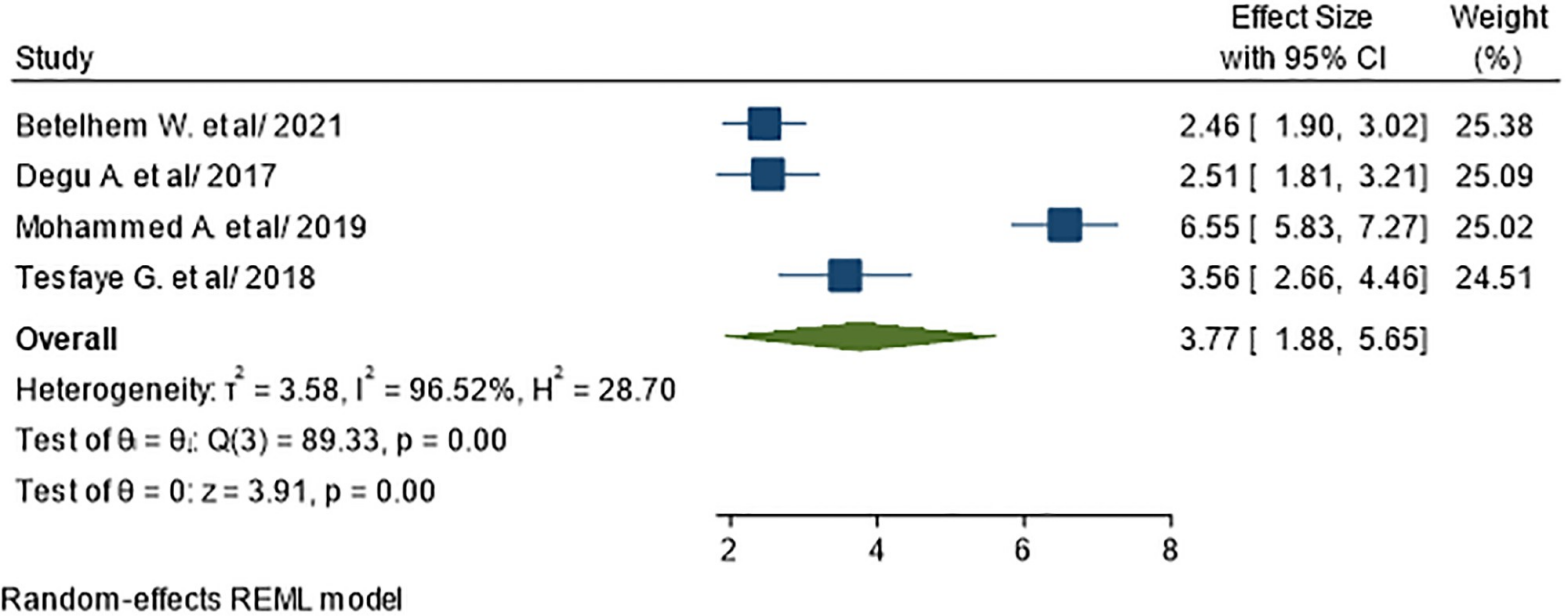
Association between female sex and UTI in diabetic patients.

We examined numerous study to assess the association of sex with UTI in diabetic patients. Accordingly, female sex was reported to be a factor with UTI by four primary studies included in this review [35, 38, 41, 43]. A total of 1071 subjects were included to analyze the association of female sex with UTI. The pooled odds ratio showed that female diabetics were 3.77 times more likely to develop UTI than males with diabetics (AOR = 3.77; 95% CI: 1.88, 5.65), I^2^ = 96.52%, P<0.01) (Fig 9).

### Association of educational status with UTI

**Fig 10:**
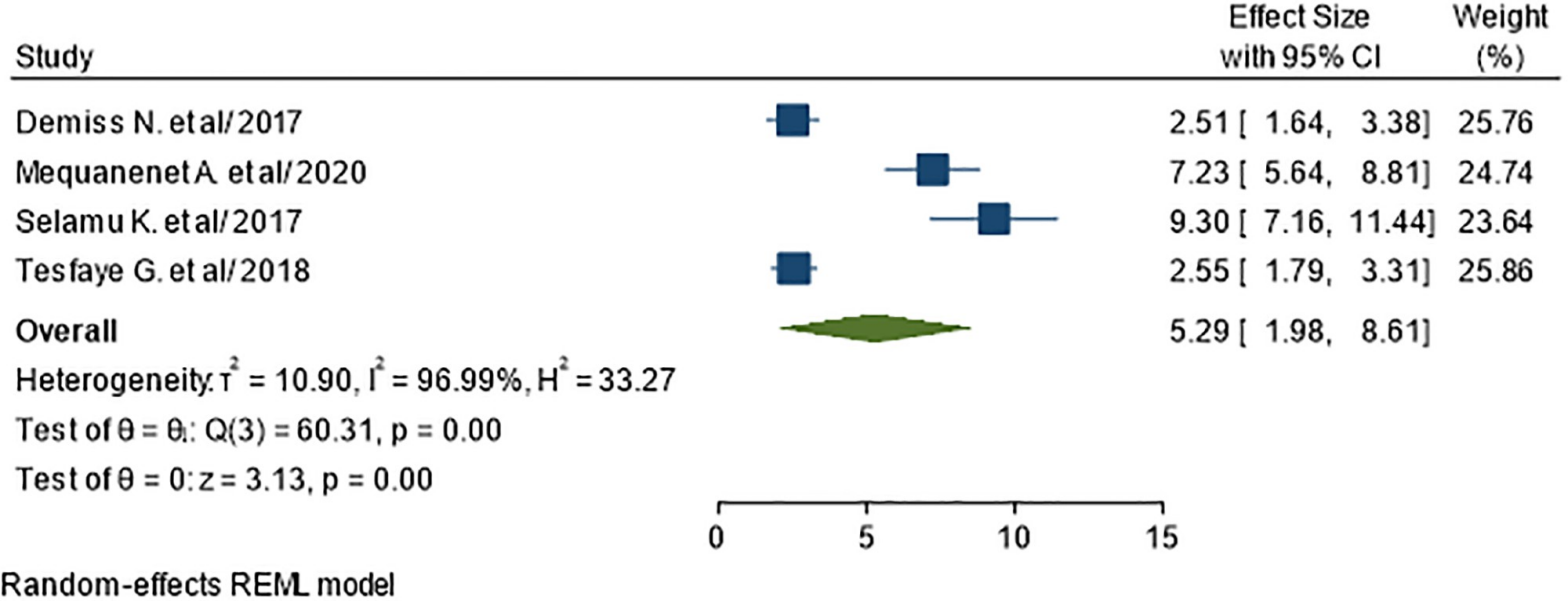
Association between educational status and UTI in diabetic patients.

To examine the association between educational status and UTI in diabetic patients, we reviewed all studies included in this systematic review. Being illiterate was identified as a significant factor associated with UTI in four primary studies included in this meta-analysis [34, 40–42]. A total of 1009 subjects were included to analyze the association between being illiterate and UTI. The odds of UTI among illiterate diabetics were 5.29 times higher than diabetics who are not illiterates (AOR = 5.29; 95% CI: 1.98, 8.61), I^2^ = 96.99%, P<0.01) (Fig 10).

### Association of previous history of UTI with current UTI

**Fig 11:**
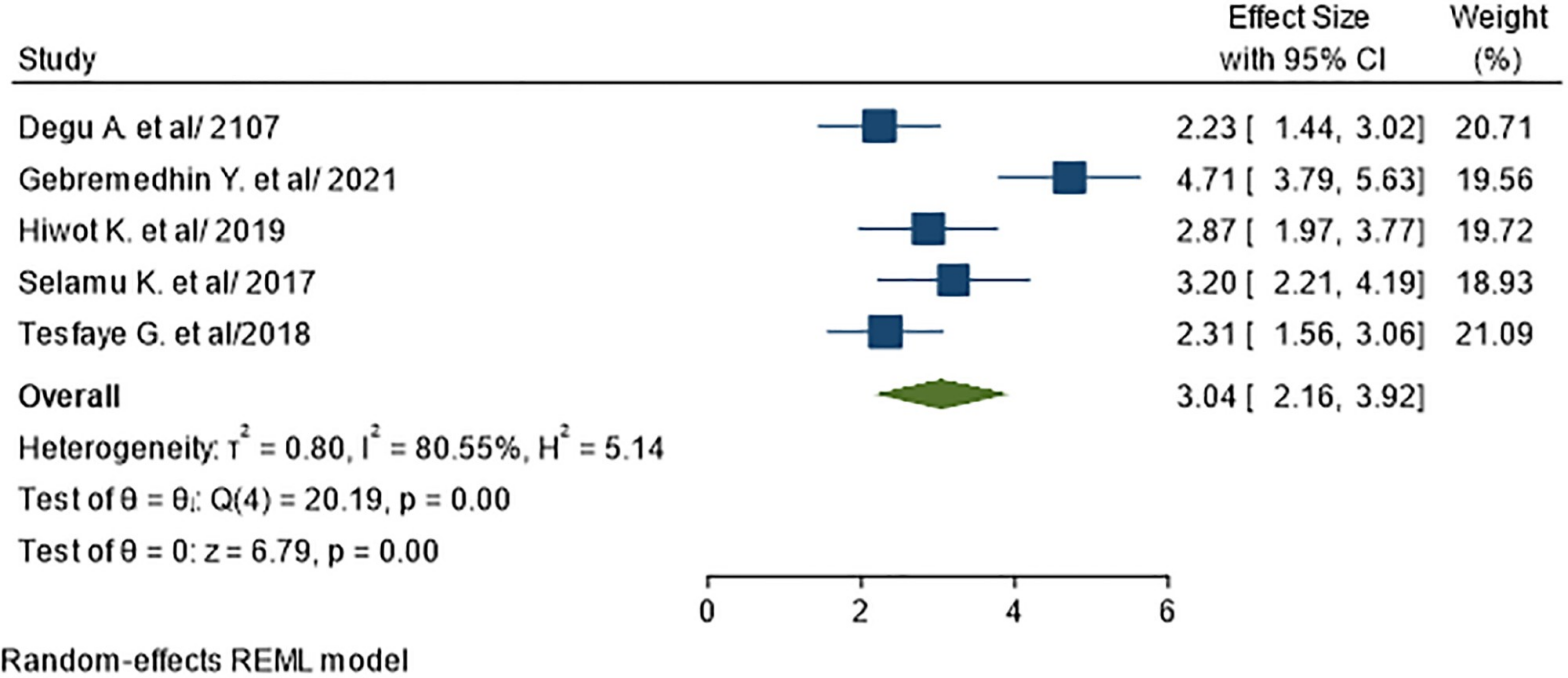
Association between prior UTI history and UTI in diabetic patients.

Five primary studies with a total subject of reported previous history of UTI as a factor for UTI in diabetic patients [31, 32, 41–43]. The pooled odds ratio showed that diabetics who had previous history of UTI were 3 times more likely to acquire UTI than their counterparts (AOR = 3.04; 95% CI: 2.16–3.92), I^2^ = 80.55%, P<0.01) (Fig 11).

### Association of high blood glucose with UTI

**Fig 12:**
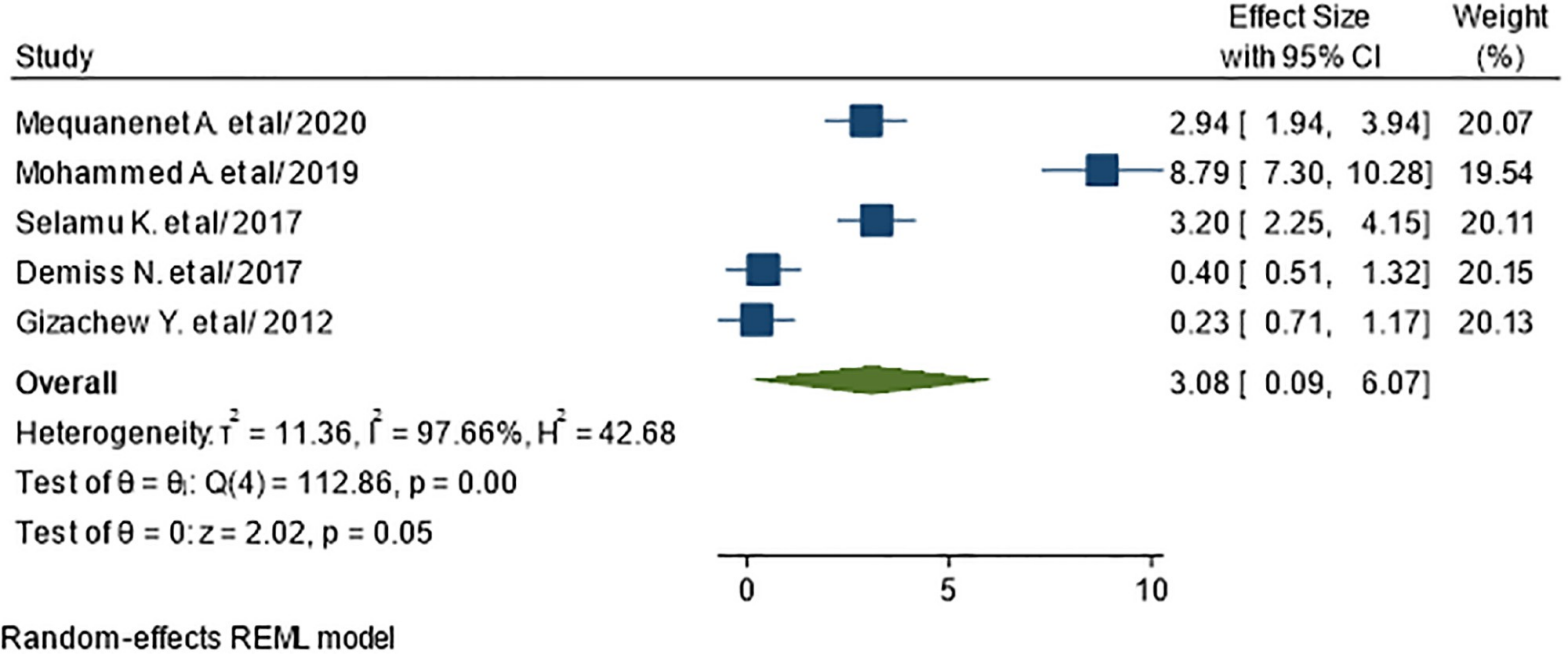
Association between high blood glucose and UTI in diabetic patients.

In order to see the association between higher level of blood glucose and UTI, five studies were included in the meta-analysis [34, 37, 38, 40, 42]. A total of 1437 participants involved in the meta-analysis. The pooled odds ratio demonstrated that higher level of blood glucose was not found as a significant factor for the occurrence of UTI in diabetic patients (AOR = 3.08; 95% CI: 0.09–6.07), I^2^ = 97.66%, P = 0.05) (Fig 12).

## Discussion

This review assessed the prevalence of urinary tract infections in diabetic patients and identified factors that influence prevalence estimates among studies. About 14 studies with 3773 research participants were included in this systematic review and meta-analysis. All studies were observational (cross-sectional) in nature, and the study participants were chosen using a simple random, systematic random, and convenience sampling approach. The sample size for each study ranged from 179 to 422. Furthermore, meta-regression was performed to identify the potential sources of heterogeneity.

In our study, we included 14 studies with 3773 subjects. The pooled prevalence of UTI among diabetes was 15.97% (95% CI: 12.72–19.23; I^2^ = 87.85%). The reason for such high prevalence of UTI in diabetic patients, the researchers inferred that the relation between UTI and diabetes was associated with poor circulation, decreased immune system due to reduced ability of white blood cells to fight infections, poor contractions of the bladder leading to bladder dysfunction are some of the contributing factors leading to increased cases of UTI in diabetics [44–46].

The result of our study is consistent with a large cross-sectional study conducted in Portugal (16.2%) [47]. The prevalence of UTI among diabetes in this systematic review and meta-analysis is higher than a systematic review and meta-analysis conducted in Iran (11.5%) [48]. In comparison, the prevalence is lower than the study results in Saudi Arabia (25.3%) [49]. The potential reason for this differences might be due to the different lifestyle of populations, a number of studies included in the analysis and the study settings.

A variety of factors were linked to UTI among diabetes in this systematic review and meta-analysis. Results by this meta-analysis suggested that sex, level of education and having history of UTI are the predictors of UTI. Hence, female diabetes are 3.77 times more likely than their counterparts to develop UTI. This result is in line with the study conducted in Saudi Arabia [49], China [50], Kuwait [51], and the U.S [52]. The difference between genders could be explained by the fact that, due to anatomical differences of the urinary tract, usually the prevalence of UTIs in women is higher than men. The higher infection rate of UTI among females in our study may be due to decrease of normal vaginal flora (Lactobacilli), less acidic pH of vaginal surface, poor hygienic condition, short and wide urethra and proximity to anus.

In the current study, UTI was more common among diabetic patients who were illiterate. This difference is due to the fact that diabetes self-management education is critical for preventing infections particularly UTI, blood glucose control, and overall self-care, and literates should have a greater understanding and ability regarding good control of blood glucose, self-care methods and early health seeking in their follow up to avoid urinary tract infections. This study demonstrated that previous history of UTI is associated with increased level of UTI in diabetic patients. This study shows that diabetes patients who had previous history of UTI is 3 times more likely to catch UTI than diabetes without previous history of UTI. The potential reason might be patients with previous history of UTI appear to be more vigilant in seeking medical care when UTI symptoms occurred. This finding was consistent with study in U.S [52].

We found differences in the prevalence of UTI in diabetic patients according to the regions with SNNP having the highest prevalence (19.2%) and Addis Ababa having the lowest prevalence (14.02%). This difference might be due to differences in lifestyle and health care-seeking behavior. Addis Ababa is the capital of Ethiopia, which is have a relatively better educational and health institutions which in-turn improves the resident’s quality of life and health than other regions in the country. Moreover, we found that the prevalence computed from studies done in and after 2018 (17.98%) was higher from studies conducted before 2018 (14.04%). This might be attributed to the difference between the advancement of diagnostic tools and increased accessibility to the health facilities which could result in better and higher number of UTI diagnosis.

This systematic review has strengths and weaknesses. We used robust search algorithms with to pull studies from multiple databases. We have estimated the pooled prevalence of UTI in diabetic patients, which is very important for preventative public health. In addition, risk factors of urinary tract infection were identified. There are some limitations in this meta-analysis. First, the heterogeneity shown between the different published studies and that may be due to differences in data collection as well as the difference in the sample size. Two, articles were restricted to only being published in the English language. Three, all included studies were cross-sectional, which might affect the outcome variable because of other confounding factors.

## Conclusion

In conclusion, our study demonstrates that the prevalence of urinary tract infection is higher. Besides, the pooled prevalence of UTI varies based on the regions and publication year. Being female, being illiterate, having previous history of UTI were predictors of urinary tract infection. Since UTI in diabetic patients has serious medical and public health consequence, screening of UTI in diabetic patients and early initiation of treatment should become a public health priority. We also recommend that public health programs in SNNP and other regional areas pay particular attention to combating the growing prevalence of UTI-DM comorbidity.

## Supporting information

S1 Checklist PRISMA checklist. (DOCX)

S1 Table Methodological quality assessment of included studies using Joanna Brigg’s Institute quality appraisal criteria scale (JBI). The eight item questions assessing inclusion criteria, study setting and participant, exposure measurement, objectives, confounder, statically analysis, outcome measurement and dealing confounder were used.
(DOCX)

S2 Table Risk of bias assessment for the included studies. The ten item questions of which four items assess external and six items assess internal validity were used.
(DOCX)

S1 Dataset The minimal anonymized data set. (XLSX)
