# Supplementary material for: Prevalence of urinary tract infections and risk factors among diabetic patients in Ethiopia, a systematic review and meta-analysis
Source: PLoS One. 2023 Jan 17;18(1):e0278028. doi: 10.1371/journal.pone.0278028 (PMC9844928; doi:10.1371/journal.pone.0278028)
Supplement: S1 Table — The eight item questions assessing inclusion criteria, study setting and participant, exposure measurement, objectives, confounder, statically analysis, outcome measurement and dealing confounder were used. (DOCX) [file pone.0278028.s002.docx]

S 2 Table. Quality assessment for the included Studies

| Item | Clearly defined inclusion | Describe study setting and participant | Valid and reliable exposure measurement | Objective and standard criteria for measurement | Identified confounder | Strategies to deal with confounders | Valid and reliable outcome measurement | Appropriate statically analysis | No of ‘yes’ ‘ |
| --- | --- | --- | --- | --- | --- | --- | --- | --- | --- |
| Yeshitela.B et al | Yes | Yes | No | Yes | Yes | No | Yes | Yes | 6/8=75 |
| Feleke Y. et al | Yes | Yes | Yes | Yes | No | No | Yes | Yes | 6/8=75 |
| Betelhem W. et al | Yes | Yes | No | Yes | Yes | No | Yes | Yes | 6/8=75 |
| Degu A. et al | Yes | Yes | No | Yes | Yes | Yes | Yes | Yes | 7/8=87.5 |
| Demiss N. et al | Yes | Yes | No | Yes | Yes | Yes | Yes | Yes | 7/8=87.5 |
| Gebremedhin Y. et al | Yes | Yes | Yes | Yes | Yes | No | Yes | Yes | 7/8=87.5 |
| Gizachew Y. et al | Yes | Yes | Yes | Yes | No | No | Yes | Yes | 6/8=75 |
| Hiwot K. et al | Yes | Yes | No | Yes | Yes | Yes | Yes | No | 6/8=75 |

| Mekuanenet A. et al | Yes | Yes | Yes | Yes | No | Yes | Yes | No | 6/8=75 |
| --- | --- | --- | --- | --- | --- | --- | --- | --- | --- |
| Mohammed A. et al | Yes | yes | No | Yes | Yes | Yes | Yes | Yes | 7/8=87.5 |
| Seble W. et al | Yes | Yes | Yes | No | Yes | No | Yes | Yes | 6/8=75 |
| Selamu K. et al | Yes | Yes | Yes | Yes | Yes | Yes | Yes | No | 7/8=87.5 |
| Tesfaye G. et al | Yes | Yes | Yes | Yes | Yes | Yes | No | Yes | 7/8=87.5 |
| Aley M. et al | Yes | Yes | No | Yes | No | Yes | Yes | Yes | 6/8=75 |
