# Supplementary material for: Prevalence of urinary tract infections and risk factors among diabetic patients in Ethiopia, a systematic review and meta-analysis
Source: PLoS One. 2023 Jan 17;18(1):e0278028. doi: 10.1371/journal.pone.0278028 (PMC9844928; doi:10.1371/journal.pone.0278028)
Supplement: S2 Table — The ten item questions of which four items assess external and six items assess internal validity were used. (DOCX) [file pone.0278028.s003.docx]

S 3 Table. Risk of bias of assessment for the cross-sectional studies

| Item | External validity | | | | Internal validity | | | | | |  | |
| --- | --- | --- | --- | --- | --- | --- | --- | --- | --- | --- | --- | --- |
|  | Representativeness s of the target population | Representativeness s of the sampling frame | Radom samplin g or census | Minimal responses e bias | Data were collect d directly | Acceptable e case definition used in the study | Valid and reliable measurement t | The same mode of data collection n for all study subject | Appropriate e length of prevalence period for parameter of interest | Appropriate numerators and denominator s of interest | No of yes | **Summ ary of risk of bias** |
| Yeshitela.B et al | Yes | Yes | No | Yes | Yes | No | Yes | Yes | Yes | Yes | 8 | Low-  risk |
| Feleke Y. et al | Yes | Yes | Yes | Yes | No | No | Yes | Yes | Yes | Yes | 8 | Loiw risk |
| Betelhem W. et al | Yes | Yes | No | Yes | Yes | No | Yes | Yes | Yes | Yes | 8 | Low- risk |
| Degu A. et al | Yes | Yes | No | Yes | Yes | Yes | Yes | Yes | Yes | Yes | 9 | Low – risk |
| Demiss N. et al | Yes | Yes | No | Yes | Yes | Yes | Yes | Yes | Yes | Yes | 9 | Low- risk |
| Gebremedhin Y. et al | Yes | Yes | Yes | Yes | Yes | No | Yes | Yes | Yes | Yes | 9 | Low- risk |
| Gizachew Y. et al | Yes | Yes | No | Yes | Yes | Yes | Yes | Yes | Yes | Yes | 9 | Low- risk |
| Hiwot K. et al | Yes | Yes | Yes | Yes | No | No | Yes | Yes | Yes | Yes | 8 | Low- risk |
| Mekuanenet A. et al | Yes | Yes | No | Yes | Yes | Yes | Yes | No | Yes | Yes | 8 | Low- risk |

| Mohammed A. et al | Yes | Yes | Yes | Yes | No | Yes | Yes | No | Yes | Yes | 8 | Low- risk |
| --- | --- | --- | --- | --- | --- | --- | --- | --- | --- | --- | --- | --- |
| Seble W. et al | Yes | Yes | Yes | Yes | No | No | Yes | Yes | Yes | Yes | 8 | Low-  risk |
| Selamu K. et al | Yes | yes | No | Yes | Yes | Yes | Yes | Yes | Yes | Yes | 9 | Low- risk |
| Tesfaye G. et al | Yes | Yes | Yes | No | Yes | No | Yes | Yes | Yes | Yes | 8 | Low-  risk |
| Aley M. et al | Yes | Yes | Yes | Yes | Yes | Yes | Yes | No | Yes | Yes | 9 | Low-  risk |
